# Anesthesia and Outcome of 33 Surgeries in 24 Multiple Endocrine Neoplasia Type 2A (MEN2A) Patients: A National Rare Disease Center’s Experience

**DOI:** 10.3389/fendo.2022.905963

**Published:** 2022-06-30

**Authors:** Yaohan Li, Di Jin, Le Shen, Yuguang Huang

**Affiliations:** ^1^ Department of Anesthesiology, Peking Union Medical College Hospital, Peking Union Medical College, Chinese Academy of Medicine Science, Beijing, China; ^2^ State Key Laboratory of Complex Severe and Rare Diseases (Peking Union Medical College Hospital), Beijing, China

**Keywords:** MEN2A, hemodynamics, pheochromocytoma, anesthesia, length of stay, outcome

## Abstract

**Background:**

Multiple endocrine neoplasia type 2A (MEN2A) is a rare syndrome that presents as medullary thyroid carcinoma, pheochromocytoma, and hyperparathyroidism. Experience is lacking in the anesthetic management of patients with this syndrome, particularly in those who present with pheochromocytoma receiving nonpheochromocytoma resection. We aimed to share our experience with the anesthetic management of MEN2A patients.

**Method:**

We retrospectively enrolled 24 MEN2A patients who had received different types of surgery at Peking Union Medical College Hospital from January 1, 2015, to December 31, 2021. All the medical records were reviewed and analyzed.

**Result:**

In total, 33 surgeries were performed in 24 MEN2A patients, with 20 surgeries comprising pheochromocytoma resection in 17 patients. Most of these patients who had received pheochromocytoma resection had typical hemodynamic changes during surgery and anesthesia. Regarding the other 13 nonpheochromocytoma resections in 13 patients, 10 were performed in patients without pheochromocytoma, and 3 surgeries were performed with either functional primary (1, bilateral tumor whose patient refused adrenalectomy) or metastatic pheochromocytoma (2, unresectable and malign tumors developed years after bilateral adrenalectomy). Regarding the latter 3 patients, 1 showed hypertension and tachycardia during anesthesia induction, 1 showed tachycardia during surgery and the other showed stability during surgery. Patients who had received pheochromocytoma resection (n=17) required longer postoperative hospital stays than those who had received nonpheochromocytoma resection without pheochromocytoma (n=10) (5.8 ± 1.8 vs. 4.3 ± 1.6; P = 0.031).

**Conclusions:**

Whenever MEN2A patients are diagnosed with pheochromocytoma, surgical resection of the pheochromocytoma remains the primary choice for MEN2A treatment. Nonpheochromocytoma surgeries performed with existing pheochromocytoma could be risky and require full caution and preparation.

## Introduction

Multiple endocrine neoplasia (MEN) syndrome is a hereditary disease that presents with at least two endocrine gland tumors. It is categorized into 3 types: MEN1, MEN2A, and MEN2B (1). MEN2A, the predominant phenotype of MEN2, is caused by germline mutations in the RET proto-oncogene that predispose carriers to an increased risk to development of medullary thyroid cancer (MTC), pheochromocytoma, and primary hyperparathyroidism to different degrees (2). The classic MEN2A susceptibility mutation in codon 634 is estimated at 3.7 live births per million, with 97% of patients presenting with MTC, half with pheochromocytoma, and one-fifth with primary hyperparathyroidism (3).

Surgeries such as thyroidectomy, adrenalectomy and parathyroidectomy remain the gold standard in treating MEN2A patients (4), particularly for all *RET* carriers who may undergo thyroidectomy with or without adrenalectomy and with or without parathyroidectomy depending on the patient’s condition. Currently, the guidelines for the management of MEN2A suggest that pheochromocytoma should be resected before surgical treatment of MTC or primary hyperparathyroidism (5–7). Almost all centers follow the literature concerning the indication of adrenalectomy before surgery. Thus, information is lacking concerning thyroidectomy for MTC with concomitant pheochromocytoma. Additionally, 50% of MEN2A patients are primarily diagnosed with MTC without pheochromocytoma, and 40% are diagnosed with pheochromocytoma and MTC concurrently (6). To date, only one case has been identified regarding tachycardia and hypotension in a patient with medullary thyroid cancer and undiagnosed pheochromocytoma (8). Pheochromocytoma should be of the prompt consideration or diagnosis after refractory treatment of severe hypertensive crisis during anesthesia induction of suspected cases (9).

Therefore, anesthesia for MEN2A patients who receive different types of surgeries with or without pheochromocytoma must be managed. Our experience in recent years might be helpful.

## Materials and Methods

This study reviewed the medical records of all MEN2A patients who had undergone surgeries at Peking Union Medical College Hospital, a national rare disease center, from January 1, 2015, to December 31, 2021. All the patients were numbered in the order of their first operation date, and the correlated surgeries were categorized into three groups: pheochromocytoma resection, nonpheochromocytoma resection without pheochromocytoma and nonpheochromocytoma resection with pheochromocytoma.

All the data, including patient age, sex, medical history, clinical manifestations, preoperative medication, laboratory tests, hemodynamics, complications, postoperative ICU stay and hospital stay, were collected. Laboratory tests included measurement of the 24-hour levels of urine catecholamines, metanephrine (MN), and nor-metanephrine (NMN), hemoglobin concentration and hematocrit. Preoperative medication for MEN2A patients included phenoxybenzamine and/or metoprolol for preoperative preparation. During surgery and anesthesia, anesthesiologists managed hemodynamic changes when necessary with medications that depended on the preoperative 24-hour levels of urine catecholamines. The episodes of systolic blood pressure 30% above baseline despite vasoactive drugs were recorded. Partial, total or complete bilateral adrenalectomy was performed by surgeons for different patients considering localization and size of the pheochromocytoma in the setting of MEN2A.

The lengths of postoperative ICU stay and postoperative hospital stay were presented as means and standard deviations. Group comparisons for continuous variables were performed using Student’s t test. Ethical approval for this study (Ethical Committee No. S-K1784) was provided by the Ethical Committee of Peking Union Medical College Hospital, Beijing, China.

## Results

A total of twenty-four MEN2A patients were enrolled from the medical record system; thirteen were female, and eleven were male. Sixteen MEN2A patients were primarily diagnosed with pheochromocytoma (F/M: 7/9) at an average age of 30.6 ± 7.3 y, and the other eight were primarily diagnosed with medullary thyroid carcinoma (F/M: 6/2) at an average age of 32.2 ± 9.5 y. Eight patients received two to three surgeries in addition to pheochromocytoma, and sixteen patients received only one surgery at this center. In total, thirty-three surgeries were performed under general anesthesia (**Table 1**). All 24 patients finally recovered after 33 surgeries.

**Table 1:** Primary diagnosis, demographics and surgeries of MEN2A patients.

Patient No.	Sex	Primary diagnosis	Age^a^ (y)	Surgery No.	Age^b^ (y)	BMI (kg/m^2^)	ASA	Type of Surgery
**1**	M	PHEO	26	1.1	47	26	II	Thyroidectomy
**2**	F	PHEO	20	2.1	21	19.7	III	PHEO resection
				2.2	24	20.4	II	PHEO resection
**3**	M	PHEO	43	3.1	47	22.8	III	PHEO resection
**4**	M	PHEO	28	4.1	28	22.9	III	PHEO resection
				4.2	29	20.5	I	Thyroidectomy
**5**	M	PHEO	33	5.1	33	19.6	III	PHEO resection
**6**	F	PHEO	36	6.1	46	21.6	II	Thyroidectomy
**7**	M	MTC	39	7.1	46	24.2	III	PHEO resection
**8**	F	PHEO	41	8.1	52	27.4	I	PHEO resection
**9**	F	PHEO	32	9.1	37	23.4	III	PHEO resection
				9.2	37	22.7	II	Thyroidectomy
**10**	F	MTC	40	10.1	42	20.7	III	PHEO resection
				10.2	44	22.4	III	PHEO resection
**11**	F	MTC	21	11.1	22	22.3	I	Thyroidectomy
				11.2	24	25.2	III	PHEO resection
**12**	F	MTC	25	12.1	35	19.9	II	PHEO resection
				12.2	35	20.4	II	Thyroidectomy
				12.3	37	19.9	II	PHEO resection
**13**	F	PHEO	42	13.1	49	21.5	II	Thyroidectomy
**14**	M	MTC	34	14.1	39	25.2	III	Thyroidectomy
**15**	F	MTC	48	15.1	57	21.8	III	PHEO resection
				15.2	58	22.1	II	Mediastinal mass resection
**16**	F	PHEO	25	16.1	30	24.4	II	Cesarean Section
**17**	M	PHEO	27	17.1	27	28.4	III	PHEO resection
**18**	M	PHEO	23	18.1	24	21	III	PHEO resection
**19**	M	PHEO	28	19.1	32	27.6	III	PHEO resection
				19.2	33	25.9	II	Lumbar spine surgery
**20**	F	MTC	26	20.1	59	23.5	III	PHEO resection
**21**	M	PHEO	28	21.1	28	29.4	III	PHEO resection
**22**	M	PHEO	36	22.1	60	23.6	II	Thyroidectomy
**23**	F	PHEO	21	23.1	26	19.1	III	Thyroidectomy
**24**	F	MTC	25	24.1	33	18.8	III	PHEO resection

Age^a^, age at primary diagnosis; Age^b^, age at surgery; BMI, body mass index; ASA, American Society of Anesthesiologists physical class; M, male; F, female; PHEO, pheochromocytoma; MTC, medullary thyroid carcinoma.

For group categorization, seventeen patients received twenty pheochromocytoma resections, ten patients without pheochromocytoma received ten nonpheochromocytoma resections, and three patients with pheochromocytoma received three nonpheochromocytoma resections.

### Pheochromocytoma Resection in MEN2A

**Figure 1:**
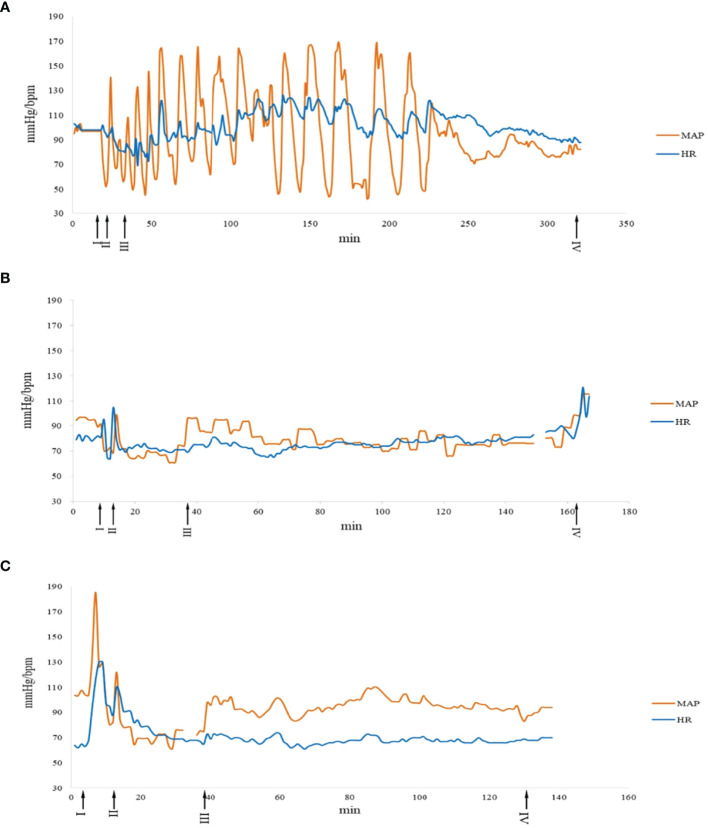
Intraoperative hemodynamic changes of three different types of surgeries in MEN2A patients. A: surgery No. 24.1, B: surgery No. 1.1, C: surgery No. 22.1. I: Anesthesia induction. II: Endotracheal intubation. III: Operation started. IV: Operation finished. The curve in break means the data lost. MAP, mean arterial pressure; HR, heart rate.

Seventeen MEN2A patients received twenty pheochromocytoma resections with all pheochromocytoma diameters > 3 cm, and they all received 2 to 4 weeks of routine preoperative medication preparation (**Table 2**). From general anesthesia induction to the end of the surgery, four surgeries (4/20) for four patients (4/17) demonstrated at least one episode of SBP > 180 mmHg, and all twenty surgeries for seventeen patients demonstrated at least one episode of SBP > 140 mmHg (**Figure 1A**). Nine surgeries (9/20) for eight patients (8/17) exhibited at least one episode of SBP 30% above baseline (**Table 2**). After surgery, all the patients returned to the ICU. The four patients who exhibited hypertensive crisis during surgery (SBP > 180 mmHg) all had typical clinical manifestations of pheochromocytoma, with a tumor diameter > 5 cm on computed tomography. The average lengths of postoperative ICU stay and postoperative hospital stay were 26.9 ± 9.8 h and 5.8 ± 1.8 d, respectively (**Table 3**).

**Table 2:** Clinical features and outcomes of 20 pheochromocytoma resection surgeries in 17 MEN2A cases.

Patient No.	Surgery No.	High 24 h urinary CA	High NMN/MN	Preoperative medication (alpha blockade)	Intraoperative SBP>30%	Length of postoperative ICU stay (h)	Length of postoperative hospital stay (d)
**2**	2.1	+	+	+	+	25	8
	2.2	–	+	+	+	24	5
**3**	3.1	+	N/A	+	–	20	4
**4**	4.1	–	N/A	+	–	23	5
**5**	5.1	+	N/A	+	–	48	7
**7**	7.1	+	N/A	+	–	24	7
**8**	8.1	–	N/A	+	–	28	7
**9**	9.1	+	N/A	+	+	26	8
**10**	10.1	+	–	+	–	24	8
	10.2	+	N/A	+	–	24	5
**11**	11.2	–	N/A	+	+	27	5
**12**	12.1	–	N/A	+	+	18	7
	12.3	–	N/A	+	–	23	3
**15**	15.1	+	N/A	+	–	53	6
**17**	17.1	+	N/A	+	–	23	3
**18**	18.1	+	+	+	–	24	4
**19**	19.1	+	+	+	+	22	6
**20**	20.1	–	–	+	+	20	4
**21**	21.1	+	+	+	+	17	5
**24**	24.1	+	+	+	+	45	9

CA, catecholamine; NMN, nor-metanephrine; MN, metanephrine; SBP>30%, systolic blood pressure 30% above baseline; ICU, intensive care unit; +, yes; -, no; N/A, not available.

**Table 3:** Length of ICU stay and postoperative hospital stay after different types of surgery in MEN2A patients.

Type of surgery	n	Length of postoperative ICU stay (h)	Length of postoperative hospital stay (d)
**PHEO resection**	20	26.9±9.8	5.8±1.8
** 24 h urinary CA positive**	13	28.8±11.6	6.2±1.9
** 24 h urinary CA negative**	7	23.3±3.5	5.1±1.5
**Non-PHEO resection without PHEO**	10	0	4.3±1.6*
**Non-PHEO resection with PHEO**	3	164.7±230.2	16.0±18.2

PHEO: pheochromocytoma, CA: catecholamine, ICU: intensive care unit, *: p<0.05 compared with the PHEO resection group.

Patients who received pheochromocytoma resection with elevated preoperative 24 h urinary catecholamine (n=13) needed longer lengths of the postoperative ICU stay (28.8 ± 11.6 h vs. 23.3 ± 3.5 h, P = 0.132) and postoperative hospital stay (6.2 ± 1.9 d vs. 5.1 ± 1.5 d, P = 0.231) than the others with normal 24 h urinary catecholamine (n=7), despite no statistical significance.

### Nonpheochromocytoma Resection in MEN2A Patients Without Pheochromocytoma

Ten MEN2A patients received ten nonpheochromocytoma resections before the occurrence of pheochromocytoma or after the resection of diagnosed pheochromocytoma (**Table 4**). Three required preoperative antihypertensive medication; none demonstrated an episode of SBP 30% above baseline (**Table 4**, **Figure 1B**). All the patients recovered full consciousness after surgery without transfer to the ICU. The average length of postoperative hospital stay was 4.3 ± 1.6 d (**Table 3**).

**Table 4:** Clinical features and outcomes of non-pheochromocytoma resection surgeries performed without the condition of pheochromocytoma in 10 MEN2A cases.

Patient No.	Surgery No.	High 24 h urinary CA	High NMN/MN	Preoperative medication (alpha blockade)	Intraoperative SBP>30%	Length of postoperative ICU stay (h)	Length of postoperativehospital stay (d)
**1**	1.1	N/A	N/A	N/A	–	0	6
**4**	4.2	N/A	N/A	N/A	–	0	2
**6**	6.1	–	–	N/A	–	0	3
**9**	9.2	N/A	N/A	N/A	–	0	3
**11**	11.1	N/A	N/A	N/A	–	0	4
**12**	12.2	N/A	N/A	+	–	0	6
**13**	13.1	N/A	N/A	N/A	–	0	4
**15**	15.2	N/A	N/A	N/A	–	0	3
**16**	16.1	–	N/A	+	–	0	6
**19**	19.2	–	+	+	–	0	6

CA, catecholamine; NMN, nor-metanephrine; MN, metanephrine; SBP>30%, systolic blood pressure 30% above baseline. ICU, intensive care unit; +, yes; -, no; N/A, not available.

### Nonpheochromocytoma Resection in MEN2A Patients With Pheochromocytoma

Three MEN2A patients with either primary or metastatic pheochromocytoma received three nonpheochromocytoma resection surgeries (**Table 5**). They all received pheochromocytoma function examinations before the non-pheochromocytoma resection surgeries. Patient No. 14 had no hypertension or episodic headache, sweating and tachycardia. His preoperative examinations showed normal norepinephrine (NE, 37.69 ug/24h, 16.69-40.65 ug/24h), normal epinephrine (E, 4.19 ug/24h, 1.74-6.42ug/24h), elevated dopamine (DA, 335ug/24h, 120.93-330.59 ug/24h), elevated NMN (2109.00 ug/24h, 0-1464 ug/24h) and elevated MN (520.00 ug/24h, 0-394 ug/24h) in 24h urinary sample. Patient No. 22 had hypertension but no episodic headache, sweating or tachycardia. His preoperative examinations showed elevated NMN (1.43 nmo1/L, 0-0.9 nmo1/L) and elevated MN (2.08 nmo1/L, 0-0.5 nmo1/L) in blood sample. Patient No. 23 had hypertension and episodic headache and tachycardia. His preoperative examinations showed elevated NE (1721.1 ug/24h, 0-76.9 ug/24h), elevated E (69.9 ug/24h, 0-11 ug/24h), and elevated DA (840.9 ug/24h, 0-459.9 ug/24h) in 24h urinary sample, with elevated NMN (51.13 nmo1/L, 0-0.9 nmo1/L) and elevated MN (2.35 nmo1/L, 0-0.5 nmo1/L) in blood sample. All three patients received preoperative medication preparation for existing hypertension and radical thyroidectomy for MTC. During surgery (1/3) for patient No. 22 (1/3), an episode of SBP 30% above the baseline occurred (**Table 5**). Patient No. 22 temporarily showed hypertension and tachycardia after induction (**Figure 1C**). They all maintained stable hemodynamic parameters during surgery, and both patients No. 14 and No. 22 were transferred to the ICU immediately after surgery. Patient No. 23, who recovered full consciousness after surgery without transfer to the ICU, experienced pheochromocytoma crisis and catecholamine cardiomyopathy on the first day postoperatively. She was then transferred to the ICU and finally recovered after a 430-hour ICU stay and 37-day postoperative hospital stay (**Table 5**). The average lengths of postoperative ICU stay and postoperative hospital stay were 164.7 ± 230.2 h and 16 ± 18.2 d, respectively (**Table 3**).

**Table 5:** Clinical features and outcomes of non-pheochromocytoma resection surgeries (thyroidectomy for medullary thyroid carcinoma, MTC) performed in 3 MEN2A patients with either functional primary or recurrent/malign pheochromocytoma.

Patient No.	Surgery No.	High 24 h urinary CA	HighNMN/MN	Preoperative medication (alpha blockade)	Intraoperative SBP>30%	Length of postoperative ICU stay (h)	Length of postoperative hospital stay (d)
14*	14.1	+	+	+		19	7
22**	22.1	N/A	+	+	+	45	4
23**	23.1	+	+	+		430	37

CA, catecholamine; NMN, nor-metanephrine; MN, metanephrine; SBP>30%, systolic blood pressure 30% above baseline. ICU, intensive care unit; +, yes; -, no; N/A, not available.

*; presence of functional bilateral pheochromocytoma but the patient and her family preferred radical thyroidectomy for MTC before adrenalectomy for the symptoms and lymphatic metastasis of MTC.

**; patients with previous history of bilateral pheochromocytoma resection surgeries admitted with unresectable pheochromocytoma (functional; recurrent; and malign) and progression of MTC.

## Discussion

The clinical signs of pheochromocytoma due to excess catecholamine release include hypertension, headache, palpitation, and sweating (10). Surgical resection is the main therapy for patients with pheochromocytoma, and preoperative medications, including alpha blockade, are beneficial for patients to reverse blood vessel contraction and reduce the risk of significant hypotension after pheochromocytoma resection. However, intraoperative hemodynamic instability remains unavoidable in patients with pheochromocytoma and may be associated with tumor size and high levels of urinary catecholamine.

The onset age of pheochromocytoma in MEN2A patients is much younger than that in nonsyndromic pheochromocytoma and paraganglioma patients in our center (11, 12). Regarding MEN2A patients, either adrenal-sparing surgery or total adrenalectomy could be therapeutic for phaeochromocytoma depending on the patient’s condition, surgeon and institution (13). Foo CW et al. reported a case of MEN2A syndrome that had undergone bilateral laparoscopic adrenalectomy, and they found that the intraoperative hemodynamic instability was much more pronounced than that in other pheochromocytoma patients (14). At our center, the proportion of patients who experienced at least one episode of SBP > 30% was 59.6% in nonsyndromic pheochromocytoma or paraganglioma resections (11), a value much higher than that in MEN2A pheochromocytoma resections (9/20, 45%). The reason may be the smaller tumor size and that only unilateral pheochromocytoma resection was preferable. A pheochromocytoma diameter larger than 5 cm is an independent risk factor for intraoperative hemodynamic instability (11).

Another retrospective study including 61 patients who had undergone pheochromocytoma resection concluded that intraoperative hypertension episodes were similar in MEN2A patients to those in nonsyndromic pheochromocytoma patients, with postoperative hypotension less frequent and severe (15). However, for MEN2A patients undergoing pheochromocytoma resection, preoperative medications, including phenoxybenzamine or other selective a1-adrenergic blockers, are necessary, and intraoperative hemodynamic instability can be predicted according to their clinical manifestations, laboratory examination and tumor size (11, 15, 16).

Nearly 10% of MEN2A patients die because of medullary thyroid carcinoma (17). The treatment for MTC is a surgical procedure, with the later the tumor stage and the worse the prognosis (18). Therefore, for such patients, thyroidectomy for hereditary C cell disease and prophylactic total thyroidectomy may be beneficial (19). Most MEN2A patients present with MTC or are primarily diagnosed with MTC; therefore, most thyroidectomies are performed before or without the clinical diagnosis of pheochromocytoma. Our experience showed stable intraoperative hemodynamic parameters of MEN2A patients without pheochromocytoma who had received nonpheochromocytoma resections, and preoperative medication might not be necessary.

Pal R et al. reported a case of metastatic pheochromocytoma in MEN2A in which the patient had received thyroidectomy accompanied by suspected metastatic pheochromocytoma after adequate preoperative preparation (20).

It’s still very risky for MEN2A patients to receive nonpheochromocytoma resection surgeries with either primary or metastatic pheochromocytoma, as could be even more risky in some extremely urgent circumstances without adequate preoperative evaluation and medications. Three patients at our center had received thyroidectomy for MTC accompanied by functional primary or metastatic pheochromocytoma. Regarding these three cases, for patient No. 14, bilateral pheochromocytomas were found in hospital during MTC treatment, but the patient and her family preferred radical thyroidectomy for MTC before adrenalectomy for the symptoms and lymphatic metastasis of MTC. For patient No. 22 and No. 23, they both received bilateral pheochromocytoma resection surgeries years ago. Unfortunately, both exhibited multiple pheochromocytoma metastasis, resulting in the inability to achieve radical pheochromocytoma resection. With the progression of MTC and correlated pathophysiological changes, they both received radical thyroidectomy with the metastatic pheochromocytomas.

Despite adequate preoperative medication preparation and less frequent hemodynamic instability than pheochromocytoma resection, they needed a similar or even longer length of stay in the ICU after the surgery. Patient No. 23 experienced severe catecholamine cardiomyopathy because of postoperative excessive catecholamine release for unknown reasons. Their preoperative 24h urinary CA levels and NMN/MN levels in blood sample or 24h urinary sample could be a predictor for their postoperative outcome.From our center’s experience in pheochromocytoma and paraganglioma patients, a maximum resting HR ≥115 beats/min, a maximum resting systolic BP ≥180 mmHg, a blood glucose level ≥8.0 mmol/L, several symptoms and signs ≥3, and an onset age ≤40 years were predictive factors for catecholamine-induced cardiomyopathy (12).

As a retrospective single-center cohort analysis, only the data of 24 MEN2A patients with 33 surgeries were collected and categorized. Statistical analyses were not very confident because of the limited sample size. The RET genetic analysis is essential and obligatory in MEN2A cases and in all cases with clinical diagnosis of MTC or pheochromocytoma. However, only few patients had received this test in our center (data not available), since most of them were referred from other centers and some of them refused a genetic analysis. Most of our clinical practice protocols on MEN2A patients were based on our much larger cohort of pheochromocytoma and paraganglioma patients. MEN2A is a rare disease, and its intraoperative anesthesia management requires further study with collaboration from different specialties and centers.

## Conclusions

Based on clinical experience in the perioperative management of pheochromocytoma and paraganglioma, a similar anesthesia protocol also works on pheochromocytoma resection in MEN2A patients. All other nonpheochromocytoma resections could be scheduled and performed safely before the diagnosis of pheochromocytoma or after the resection of diagnosed pheochromocytoma. Nonpheochromocytoma resection combined with existing functional pheochromocytoma requires considerable caution and care from an experienced anesthesia or perioperative medical team.

## Data Availability Statement

The raw data supporting the conclusions of this article will be made available by the authors, without undue reservation.

## Ethics Statement

The studies involving human participants were reviewed and approved by Peking Union Medical College Hospital. The patients/participants provided their written informed consent to participate in this study.

## Author Contributions

YL collected and analyzed the data. YL and DJ wrote the manuscript. LS designed the study. LS and YH reviewed the manuscript and approved it for publication.

## Funding

This work was supported by the Education Reform Project Foundation for the Central Universities of Peking Union Medical College (2020zlgc0105).

## Conflict of Interest

The authors declare that the study was conducted in the absence of any commercial or financial relationships that could be construed as a potential conflict of interest.

## Publisher’s Note

All claims expressed in this article are solely those of the authors and do not necessarily represent those of their affiliated organizations, or those of the publisher, the editors and the reviewers. Any product that may be evaluated in this article, or claim that may be made by its manufacturer, is not guaranteed or endorsed by the publisher.
